# Body Piercing with a Metallic Tongue Stud Resulting in Ineffective Implantable Cardioverter-defibrillator Shocks: “Heart to Mouth”

**DOI:** 10.19102/icrm.2021.121105

**Published:** 2021-11-15

**Authors:** Manavotam Singh, Vijaywant Brar, Nebu Alexander, Rustin Tashayyod, Susan O’Donoghue, Seth J. Worley

**Affiliations:** ^1^Cardiac Electrophysiology MedStar Heart and Vascular Institute, MedStar Washington Hospital Center, Washington, DC, USA; ^2^MedStar Washington Hospital Center, Washington, DC, USA

**Keywords:** Failed ICD shocks, left ventricular assist devices, metal piercings

## Abstract

Left ventricular assist devices (LVADs) provide circulatory support to patients with severe left ventricular systolic dysfunction. Many such patients have a pre-existing implantable cardioverter-defibrillator (ICD) at the time of their LVAD surgery. LVAD implantation can alter the ICD lead parameters, including R-wave sensing, right ventricular capture threshold, and impedance. These changes can in turn affect the ability of the ICD to successfully treat malignant ventricular arrhythmias. In most patients who present with ineffective ICD shocks, the failed shock is assumed to be secondary to the patient’s severe cardiomyopathy. Especially, the role of physical examination in such patients is often minimized. In our patient, a thorough history-taking and history-guided physical examination led us to the root cause of the failed ICD shocks. Our patient was noted to have a metal tongue piercing, which was the likely cause of his ineffective ICD shocks. Our case highlights the importance of a comprehensive history-taking and physical examination.

## Introduction

The introduction of left ventricular assist devices (LVADs) into clinical practice over the past decade has improved the care of patients with end-stage heart failure.^1–3^ Many patients have an existing implantable cardioverter-defibrillator (ICD) at the time of LVAD implantation. LVAD implantation can alter the ICD lead parameters, including right ventricular (RV) capture threshold, RV lead impedance, and R-wave sensing.^4–6^ Reduced R-wave sensing can result in undersensing of malignant ventricular arrhythmias (VAs) and, hence, withholding of appropriate therapy, including anti-tachycardia pacing (ATP) and shocks. On the other hand, oversensing of noise generated by the LVAD can result in inappropriate ICD shocks. Furthermore, the impact of LVAD implantation on defibrillation thresholds (DFTs) has not been clearly established. To the best of our knowledge, no study has systematically looked at DFTs both pre- and post-LVAD implantation.

Recently, there have been reports of LVAD patients presenting with multiple ineffective ICD shocks.^7^ Whether shock failure is the result of LVAD implantation or the patient’s severe cardiomyopathy is unknown. The workup of post-LVAD patients with pre-existing ICDs presenting with ineffective ICD shocks is limited, especially the importance of an exhaustive history-taking and physical examination in such patients as they are considered to be of low yield. We present a case of failed ICD shocks in an LVAD patient due to the presence of a metal tongue piercing. Our case highlights the importance of a dedicated history-taking and thorough physical examination.

## Case presentation

**Figure 1:**
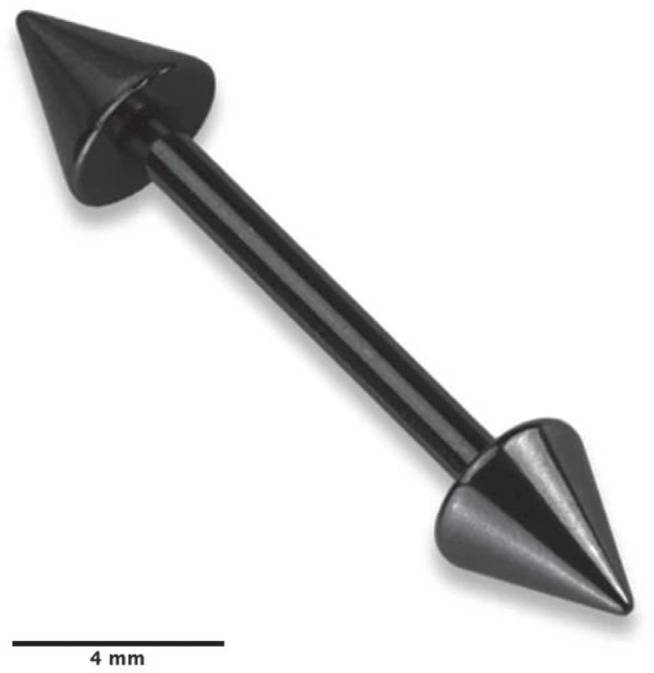
A metal tongue piercing (approximately 16 mm long, stainless steel) similar to the patient’s piercing.

**Figure 2:**
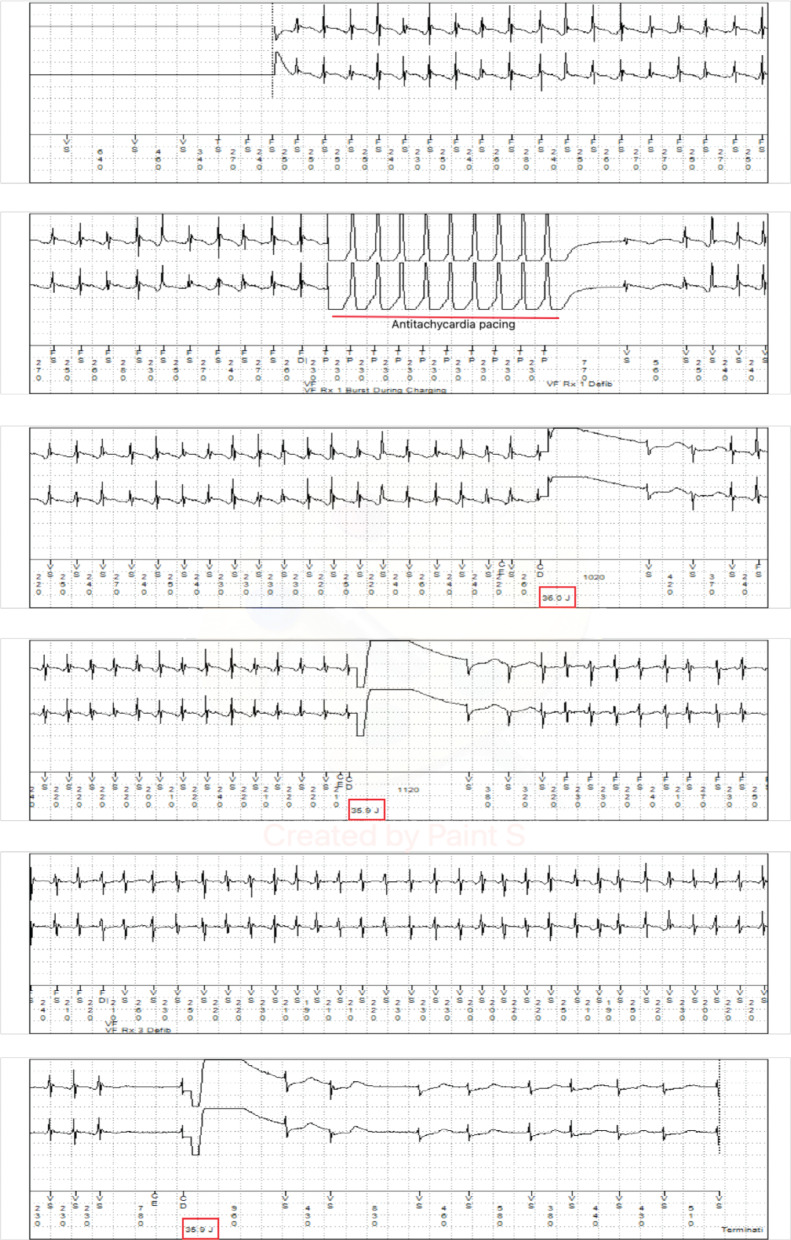
The ECGs obtained from device interrogation demonstrating an episode of sustained VT at a rate of around 240 bpm, lasting approximately 43 seconds. The patient received ATP (marked in red) followed by two failed ICD shocks. Later, he spontaneously converted to normal sinus rhythm just prior to the third shock. The shocks are marked by red boxes.

**Figure 3:**
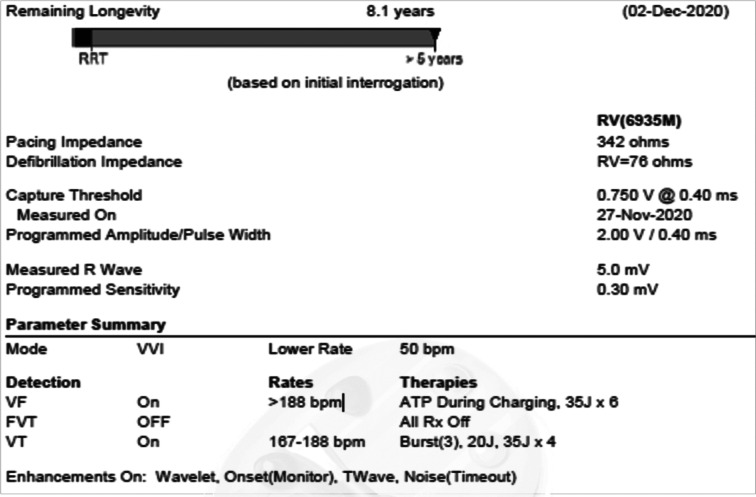
Patient’s ICD settings and lead parameters. ATP: anti-tachycardia pacing; FVT: fascicular ventricular tachycardia; RRT: recommended replacement time; RV: right ventricle; VF: ventricular fibrillation; VT: ventricular tachycardia.

A 50-year-old man with a past medical history of severe nonischemic cardiomyopathy necessitating implantation of a HeartMate 3 (HM3) LVAD (Abbott, Chicago, IL, USA) presented with multiple ineffective shocks caused by his Medtronic single-chamber dual-coil ICD **(Figure 1)**. On thorough history-taking, he described his ICD shocks as “a brick hitting my face.” The patient had no history of ICD shocks in the past and this was his first presentation for ICD shocks after LVAD implantation. He was not on any antiarrhythmic drugs at the time of presentation. On physical examination, the patient was awake and conversant with a mean arterial blood pressure of 79 mmHg, an oxygen saturation of 97% on room air, and a heart rate of 92 beats per minute (bpm). He was noted to be wearing a large metal piercing on his tongue (approximately 16 mm long, stainless steel, **Figure 1**). His lungs were clear to auscultation, and he had a normal LVAD hum over the precordium. His HM3 interrogation displayed a flow rate of 4.2 L/min, using 4 W of power at a speed of 5,400 revolutions per minute. Initial electrocardiogram demonstrated normal sinus rhythm with premature ventricular contractions. ICD interrogation demonstrated an episode of sustained ventricular tachycardia (VT) at a rate of around 240 bpm, lasting approximately 43 seconds **(Figure 2)**. The patient received ATP followed by two failed ICD shocks without termination of the VT **(Figure 3)**. He then spontaneously converted to normal sinus rhythm just prior to the delivery of the third shock (35.9 J with an impedance of 58 Ω). Initial laboratory work was significant for a serum creatinine level of 1.5 mg/dL from a baseline of 0.9 to 1.0 mg/dL, a potassium level of 4.3 mmol/L, and a magnesium level of 2.2 mg/dL. The cardiac electrophysiology (EP) team was consulted, and the patient was admitted to the cardiac intermediate care unit.

The unusual description of his ICD shock, ie, “a brick hitting his face,” and the presence of a sizable metal tongue piercing raised the possibility that his metal tongue piercing might have affected the shock effectiveness by changing the vector. Hence, we decided to perform a noninvasive programmed stimulation and test the effectiveness of his ICD shocks after removal of the metal tongue piercing. The patient was brought to the EP lab, his metal tongue piercing was removed, and his ICD was programmed to shock at 25 J, which was 10 J lower than his previously programmed shock output. Next, sustained VT was induced with a T-wave shock. The patient’s ICD appropriately detected VT and delivered a 25-J shock, which successfully terminated the rhythm. Additionally, the shock impedance after the removal of the tongue piercing was noted to be 73 Ω, which was higher when compared to 58, 66, and 67 Ω from his initial shocks **(Table 1)**.

## Discussion

The incidence of VAs in LVAD patients is high, ranging between 19% and 34% even after only 8 to 12 months post-LVAD implantation.^8^ Risk factors for VAs in such patients include electrolyte abnormalities, acidosis, hypoxemia, cardiac ischemia, etiology of the underlying cardiomyopathy, and VAs prior to the LVAD implantation.^9–11^ Interestingly, despite providing adequate hemodynamic support and offloading the left ventricle, LVADs do not reverse the underlying arrhythmogenicity.^12^

The majority of LVAD patients have ICDs implanted for primary or secondary prevention of sudden cardiac death prior to the LVAD surgery. Recently, in a large retrospective observational study of 122 LVAD patients, Galand et al.^13^ reported that 15% of the patients exhibited a greater than 50% decrease in RV sensing, 42% had a greater than 100-Ω increase/decrease in RV pacing impedance, and 20% experienced a greater than 50% increase in RV pacing threshold after LVAD implantation. Similar results have been reported by Foo et al.,^4^ Thomas et al.,^6^ and Boudghène-Stambouli et al.^14^ These changes can impact the ability of the ICD to detect and treat VAs. On the one hand, undersensing the VA may lead to no therapy being delivered by the ICD; on the other hand, the device may inappropriately deliver therapy when not indicated. Furthermore, recently, there have been increasing reports of LVAD patients presenting with multiple ineffective ICD shocks. As routine DFT testing is not performed at the time of initial ICD implantation and pre- and/or post-LVAD placement, it is not known whether elevated DFTs in such patients are due to the LVAD placement per se or simply reflect the severity of the patient’s cardiomyopathy. If LVAD placement does increase the DFTs, the mechanism(s) could be multifactorial, including: (1) vector shifts caused by the introduction of intrathoracic metal from the LVAD, ie, the LVAD itself may act as a current sink and shunt current away from the heart; (2) dislodgement of the RV lead^4^; (3) change in the orientation of the heart after LVAD implantation; and (4) use of antiarrhythmic drugs that raise DFTs.^5^

When an LVAD patient presents with ineffective ICD discharges, the ICD should be immediately disarmed, and the patient should be externally defibrillated.^15^ The subsequent evaluation of such patients should include: (1) a detailed history-taking to search for any factors that could provoke malignant VAs (heart failure exacerbation, ischemia, unusual physical/mental stress, etc.), and special attention should be paid to exclude recent initiation of any drugs that could raise DFTs; (2) a meticulous physical examination; (3) a laboratory evaluation including serum electrolytes; (4) a chest X-ray to look for appropriate placement of the ICD lead and exclude lead fracture; and (5) a comprehensive interrogation of the patient’s ICD.

In our patient, a thorough history-taking and history-guided physical examination led us to investigate any role that the metal tongue piercing may have played to result in ineffective ICD shocks. We decided to perform defibrillation testing after removal of the metal tongue piercing. Lo and behold, after the piercing was removed, the ICD was successfully able to defibrillate the patient by delivering a shock 10 J lower than the previously programmed shock output. The latter proved that the metal tongue piercing was indeed responsible for the failed ICD shocks in our patient. We hypothesize that a portion of the electric charge was shunted away from the myocardium toward the patient’s face due to the presence of a large metallic object. The latter was further supported by an increase in the shock impedance upon removal of the tongue piercing **(Table 1)**.

Our case also brings into question the safety of wearing metallic body piercings in patients with ICDs. The reduced efficacy of the ICD shocks observed in our LVAD patient could likely also be true for patients without LVADs. Further, whether the location of the metal body piercing is relevant also remains to be determined. Larger studies investigating patients with ICDs and body piercings are required.

## Conclusion

Clinicians should be aware of the potential for ineffective ICD shocks in LVAD patients. A thorough history-taking and history-guided physical examination are critical for determining the cause of failed ICD shocks in such patients. Additionally, metal piercings may result in failed ICD shocks, but this needs to be investigated in larger studies.

## Figures and Tables

**Table 1:** Ineffective Shocks Received by the Patient

Shock No.	Tongue Piercing	Energy	Successful	Shock Impedance
1	Yes	36 J	No	67 Ω
2	Yes	35.9 J	No	66 Ω
3	Yes	35.9 J	N/A	58 Ω
4	No	25 J	Yes	73 Ω

N/A: not available.
